# The characteristics of intestinal flora of IBS‐D with different syndromes

**DOI:** 10.1002/iid3.348

**Published:** 2020-09-17

**Authors:** Guanqun Chao, Shuo Zhang

**Affiliations:** ^1^ Department of General practice, Sir Run Run Shaw Hospital Zhejiang University Hangzhou Zhejiang China; ^2^ Department of Gastroenterology, The First Affiliated Hospital Zhejiang Chinese Medical University Hangzhou Zhejiang China

**Keywords:** diarrhea‐predominant irritable bowel syndrome, intestinal flora, liver‐depression and spleen‐deficiency, spleen–kidney‐yang deficiency

## Abstract

**Objective:**

To study the distribution of fecal microbiota in diarrhea‐predominant irritable bowel syndrome (IBS‐D) patients of spleen–kidney‐yang deficiency and liver depression and spleen deficiency, to make an objective foundation for dialectics of different type of IBS‐D. And to provide the clinical doctors an experimental basis for medication by regulating dysbacteriosis.

**Methods:**

We collected feces from the control group, spleen–kidney‐yang deficiency IBS‐D group, and liver‐depression and spleen‐deficiency IBS‐D group. After the extraction of fecal DNA, global DNA was isolated from every sample, and 16*S* ribosomal RNA was sequenced, and then we analyzed the results for bacteria such as Alpha diversity, community composition, LEfSe, and partial least squares discriminant analysis.

**Results:**

We compared the changes among the fecal bacteria in the intestine of the IBS‐D patients and healthy controls and found the specificity of spleen–kidney‐yang deficiency syndrome and liver‐depression and spleen‐deficiency syndrome. The control group has the highest flora diversity (control group > liver‐depression and spleen‐deficiency > spleen–kidney‐yang deficiency group). The control group, spleen–kidney‐yang deficiency group, and liver‐depression and spleen‐deficiency group are different in phylum (Actinobacteria, Fusobacteria), class (Actinobacteria, Fusobacteria), order (Enterobacteriales, Bifidobacteriales, Fusobacteriales), and family (Bifidobacteriaceae, Ruminococcaceae, Enterobacteriaceae, Acidaminococcaceae, Veillonellaceae, Fusobacteriaceae). Bifidobacteriaceae and Ruminococcaceae in the control group, Enterobacteriales, Fusobacteriales, Acidaminococcaceae, and Phascolarctobacterium in the spleen–kidney‐yang deficiency group, and streptococcus are the specific bacteria in the liver‐depression and spleen‐deficiency group. Intestinal flora disturbance is closely related to IBS‐D.

**Conclusions:**

There is a correlation between traditional Chinese medicine syndrome type and intestinal flora. The control group, the spleen–kidney‐yang deficiency group, and the liver‐depression and spleen‐deficiency group have specific bacteria.

## Graphical abstract

1. Irritable bowel syndrome (IBS) is one of the most frequent gastrointestinal disorders. Transforming growth factor‐beta 1 (TGF‐β1) is in the colonic tissue and serum of IBS rats.

2. It is suspected in our study that increased chronic renal failure and interleukin 1 beta (IL‐1β) are involved in the mechanism of IBS.

3. Further research will be done to detect the interacting and inter adjusting mechanism of IL‐1β and other cytokines in IBS patients, and further study should be done to explore the role of TGF‐β1.

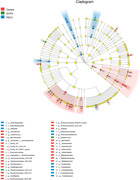

**Abbreviations**
B/E: the ratio of Bifidobacterium to Enterobacter
IBS: irritable bowel syndrome
IBS‐C: IBS with constipation
IBS‐D: IBS with diarrhea
IBS‐M: IBS with mixed bowel habits or cyclic pattern
IBS‐U: unsubtyped IBS
PLS‐DA: partial least squares discriminant analysis
TCM: traditional Chinese medicine

## 1 BACKGROUND

Irritable bowel syndrome (IBS) is one of the most common disorders among gastrointestinal disorders with an incidence of 10%–20%.^1^ Worldwide, about 5%–20% of the population is affected by the disease,^2^ while the incidence of IBS in China is about 7.26%–11.5%.^3^ The main clinical characteristics of IBS are abdominal distention, bowel habit change, and abnormal mass, accompanied by abdominal pain, symptoms persisting, or intermittent attacks,^4^ which often lack evidence to explain the symptoms by auxiliary examination.^5^ Nowadays the pathogenesis of IBS is mainly associated with nerve–endocrine–immune dysfunction, abnormal gastrointestinal dynamics, intestinal disorders, and so on.^6^, ^7^, ^8^ IBS can be classified into four types: IBS with diarrhea (IBS‐D), IBS with constipation (IBS‐C), IBS with mixed bowel habits, or cyclic pattern (IBS‐M), and unsubtyped IBS (IBS‐U) according to the stool traits.^9^ The repeated episodes of prolonged diarrhea reduce the life quality of patients, affect daily work life, and increase the financial burden of continuous medical treatment.^10^

However, a lot of medicines provide no effective therapy for IBS,^11^ while traditional Chinese medicine (TCM) has shown a more significant effect in clinical practice. IBS‐D belongs to the category of TCM "diarrhea." There was a research study^12^, ^13^, ^14^ comparing western medicine and placebo with TCM, the curative effect of Tongxieyaofang in diarrhea type of IBS showed quick action, lasting effect, stronger ability to relieve symptoms. In addition, some studies showed the effect of TCM for IBS‐D by different syndromes^15^, ^16^ and the result showed that the experimental group treated by TCM had better improvement.

In recent years, it is believed that the imbalance of intestinal flora is one of the main causes of the occurrence of IBS‐D.^17^ Studies have shown that enterobacterium increased, and bifidobacteria and lactobacillus decreased in IBS‐D.^18^ In addition, endotoxin lipid polysaccharide also has a certain effect on intestinal motility.^19^ Related research has confirmed that the number of bifidobacterium and lactobacillus have a significant increase in rats with intestinal dysfunction gavaged by Sijunzi tang.^20^ Therefore, the relationship between TCM and intestinal flora cannot be ignored. This experiment combined the TCM differentiation of IBS‐D and intestinal flora together and looked for biological links between them so as to provide a new reference standard and thinking method for the treatment system of TCM.

## 2 MATERIALS AND METHODS

### 2.1 Study design

This study applies a prospective cohort control study method, and, according to TCM criteria, IBS‐D patients are divided into the liver‐depression and spleen‐deficiency group and spleen–kidney‐yang deficiency group. The Minimum Standards of Reporting Checklist contains details of the experimental design, and statistics, and resources used in this study.

### 2.2 Participants and setting

All cases and specimens were collected in the gastroenterology department of the first hospital affiliated hospital of Zhejiang Chinese Medical University from April 2016 to September 2016.

### 2.3 Diagnostic criteria

All cases were at the ages of 18–60 years and accorded to Rome IV.

The classification and grouping were carried out according to the consensus on IBS by Chinese Medicine (Branch of Gastrointestinal Diseases, China Association of Chinese Medicine). The identification of syndromes must be present with the main symptoms and two or more accompanying symptoms.

### 2.4 Groups' division

Control group (Group C): *n* = 10.

Spleen‐kidney‐yang deficiency group (Group S (PSYX)): *n* = 10.

Liver‐depression and spleen‐deficiency group (Group L (GYPX)): *n* = 10.

### 2.5 Sample collection and processing

The stool collected in the ventilation area was quickly separated into the sterile frozen storage tube, about 1–2 g/tube, and the frozen storage tube was placed in liquid nitrogen for 4 h, and the reserve then placed at −80°C.

### 2.6 Extraction and preservation of DNA samples

The specific steps were done according to the instructions of the kit. The 200 mg stool samples were put into a 2 ml centrifuge tube. We put the centrifuge tube into a new 2 ml collection tube and added 750 μl DNA elution buffer. The concentration of genomic nucleic acid was determined by testing 1 μl extracted fecal DNA.

### 2.7 DNA samples test

We centrifuged the samples to fully blend, then took 2 μl samples to test. We observed the strip condition after conducting electrophoresis in a 2% agarose gel under 5 V/cm for 30 min.

### 2.8 Polymerase chain reaction (PCR) amplification

PCR followed the basic principles in that the low cycle number amplification was adopted and the circulation number of the single sample amplification was consistent.

### 2.9 Fluorescence quantitation

According to the results of the electropherogram of the preliminary sample quantitative determination, the PCR products were quantitatively tested by microfluorometer (type TBS380), and mixed in the corresponding proportion, respectively, according to the requirement of sequencing of each sample.

### 2.10 Construction and sequencing of MiSeq library

The viscosity of the 3ʹ end was recovered by cutting “fluorescent groups” and “termination groups.” The collected fluorescent signals were collected at the end of each round and the sequence of template DNA fragments was obtained by statistics.

### 2.11 Bioinformatics analysis

#### 2.11.1 The statistics of the original sample

In accordance with the sequence of the index in the difference between the data information of each sample, the acquired data was preserved in the form of FASTQ. Each sample of Mate Pair or Paired‐End data had two files (fp1 and fq2) with reads at the ends of the sequencing, and the sequence corresponded to the orders one by one.

#### 2.11.2 Majorizing sequence

Rogue the data of double‐end sequence data of sequencing by MiSeq though FLASH and Trimmomatic software. The allowed mismatch number of barcodes was 0, and the maximum mismatch number of primers was 2.

#### 2.11.3 OUT clustering

According to the 97% similarity, the OTU clustering was performed on the non‐repetitive sequence (without the single sequence) and the chimera was eliminated in the clustering process to obtain the representative sequence of OTU. Subsequently, the optimal sequence similar to the representative sequence in 97% was selected.

#### 2.11.4 Taxonomic analysis

RDP Classifier was performed to analyze the operational taxonomic unit (OTU) representative sequence of 97% similarity level on the QIIME platform. The results were compared with the SILVA database, and the community composition of each sample was calculated at the eight levels of domain, kingdom, phylum, class, order, family, genus, and species.

#### 2.11.5 Rarefaction curve

The majorizing sequence was randomly selected in the sequence of OTU or other taxonomic at the level of 97% similarity. The selected sequence number and the corresponding OTU number were used to construct the rarefaction curve. Diagrams were made by the R language tool.

#### 2.11.6 Alpha diversity analysis

The index of OTU in the sample was estimated by using the Chao1 algorithm in Mothur software.

#### 2.11.7 Community histogram

According to the results of taxonomic analysis, the composition of community structure at different classification levels was obtained.

#### 2.11.8 Partial least squares discriminant analysis (PLS‐DA)

The partial least square method was used to analyze the projection of OTU data structure. Diagrams were made by the R language tool.

#### 2.11.9 Linear discriminant analysis effect size

LEfSe analysis software was used to identify the characteristics of different abundances and the associated category and evaluate whether these differences were consistent with the expected biological behavior in different groups divided by taxonomy.

### 2.12 Statistical analysis

The data was represented by average ± standard deviation $\left(\bar{X}\pm s\right)$. We used statistical software such as SPSS 22.0, Usearch, Mothur, Metastat software to calculate the balance and difference in the flora data. The difference between groups was analyzed by the rank‐sum test. The difference was statistically significant at *p* < .05.

## 3 RESULT

### 3.1 General information

There were 18 males and 12 females, aged 27–60 years, with an average age of 38 years  (38.03 ± 10.28; Table 1).

**Table 1:** General information

		Diarrheal IBS group	
	Control group	Spleen–kidney‐yang deficiency group	Liver‐depression and spleen‐deficiency group
Cases	10	10	10
Sex ratio (F/M)	6/4	7/3	5/5
Age (years)	35.6 ± 8.20	42.00 ± 11.22	37.80 ± 9.65

### 3.2 DNA test

**Figure 1:**
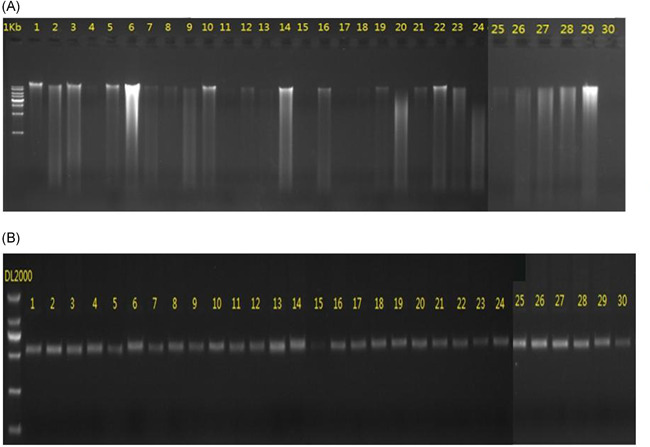
A, DNA electrophoresis and (B) identification of PCR amplification. PCR, polymerase chain reaction

A total of 30 samples were sequenced in this study. The result showed standard concentration, high purity (Table 2), and integrality (Figure 1).

**Table 2:** DNA test

Samples	Concentration (ng/μl)	OD 260/280	Sample	Concentration (ng/μl)	OD 260/280
C1	44.4	1.65	P6	48.3	1.85
C2	76.1	1.95	P7	7.1	1.46
C3	94.0	1.80	P8	12.0	1.75
C4	86.7	1.89	P9	33.7	1.91
C5	13.5	1.74	P10	98.7	1.89
C6	56.4	1.81	G1	48.5	1.94
C7	55.8	1.95	G2	114.2	1.77
C8	66.9	1.80	G3	130.9	1.73
C9	159.0	1.94	G4	9.3	2.14
C10	10.7	1.18	G5	59.7	1.78
P1	13.5	1.70	G6	282.2	1.91
P2	40.6	1.68	G7	32.2	1.83
P3	12.9	1.83	G8	18.7	1.73
P4	142.4	1.92	G9	90.8	1.97
P5	11.7	1.76	G10	56.5	1.93

### 3.3 Identification of PCR amplification

The PCR amplification product strips of 16*S* ribosomal RNA were basically clear, which met the test requirements of the Illumina MiSeq sequencing platform as expected (Figure 1).

### 3.4 Taxonomic analysis

We received 1153534 of optimization sequence (Table 3) that was based on the general principles of phylogenetics and population genetics.

**Table 3:** Effect sequence number in each sample

Samples	Sequences	Bases	Average sequence length	Shortest sequence length	Longest sequence length
C1	34225	15125396	441.939985391	418	513
C2	44250	19312551	436.441830508	358	452
C3	33965	14823742	436.441689975	391	495
C4	36272	15957067	439.927960962	396	459
C5	35588	15583375	437.882853771	305	452
C6	43489	18924058	435.145852974	407	452
C7	32533	14160779	435.274306089	417	452
C8	33932	14798189	436.113079099	282	499
C9	43106	18901684	438.493110008	324	495
C10	37062	16207455	437.306540392	418	451
P1	32407	14262715	440.112167124	358	454
P2	43921	19143992	435.873318003	385	451
P3	39923	17179483	430.315432207	301	486
P4	42319	18557457	438.513599093	358	474
P5	32034	13930344	434.861209964	412	452
P6	43246	19077058	441.128844286	358	452
P7	36109	15818667	438.081004736	335	471
P8	33658	14893230	442.487075881	358	453
P9	36103	16082240	445.454394372	422	474
P10	39557	17361667	438.902520414	291	452
G1	40032	17569830	438.894634293	319	524
G2	36956	16125332	436.338673017	400	452
G3	40432	17610801	435.565913138	407	512
G4	37130	16307897	439.210799892	419	477
G5	42286	18738104	443.127843731	358	451
G6	32323	14041815	434.421773969	418	452
G7	44566	19581067	439.372324193	385	452
G8	44421	19564525	440.434141510	420	467
G9	40111	17807265	443.949664681	343	452
G10	41578	18270802	439.434364327	396	460

### 3.5 Sequencing depth and the analysis of sample size

**Figure 2:**
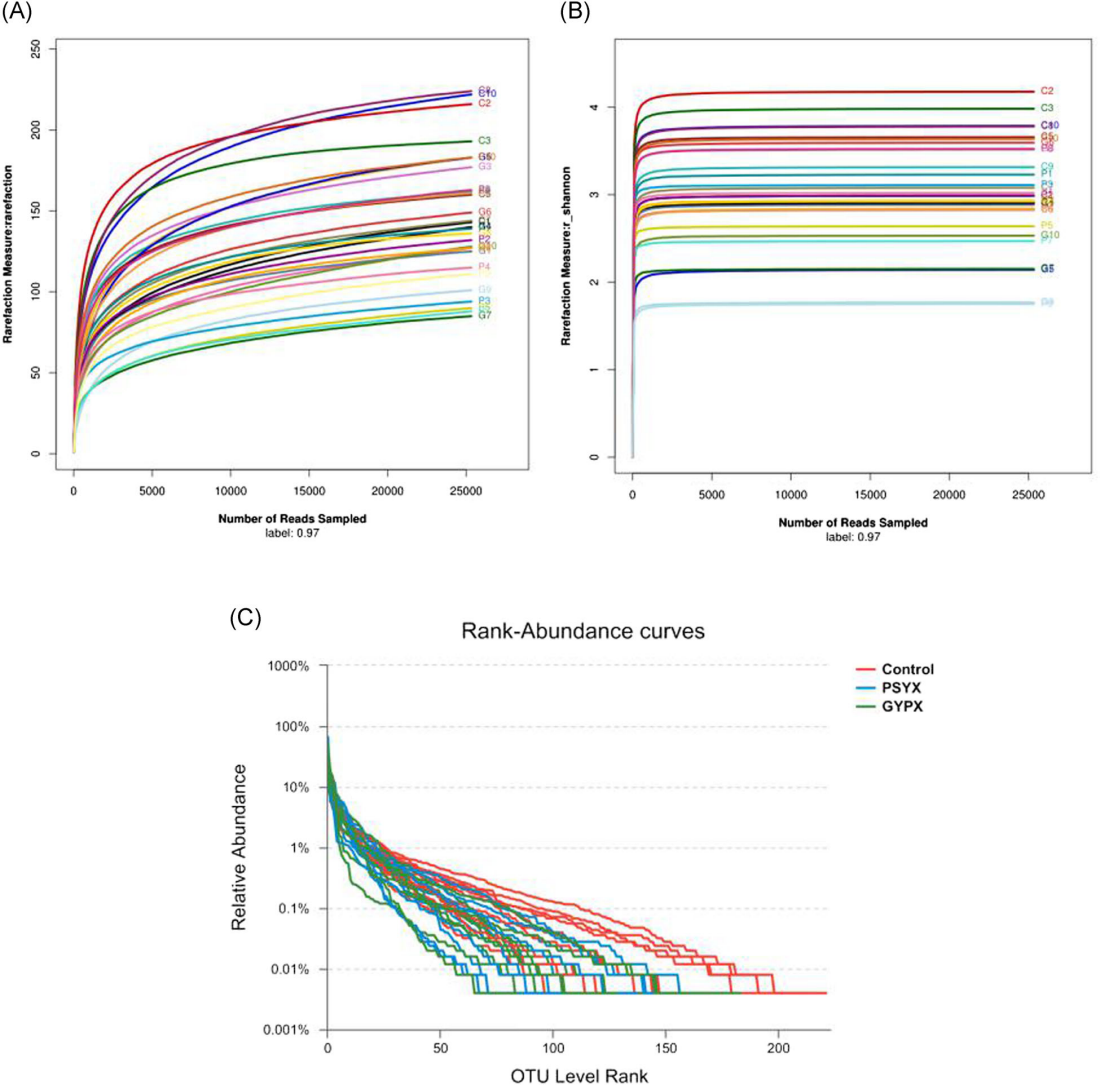
A, The rarefaction curves of the control group and the experimental groups. B, The Shannon curves of the control group and the experimental groups. C, Rank‐abundance curves of the control group and the experimental groups

The number of OTU clustering was 413, including 312 species, 164 genera, 56 family, 31 order, 18 class, 11 phyla, 1 kingdom, and 1 domain. In this study, all sample sequences were divided by OTU classification under the conditions of 97% similarity. The study could be carried out with reasonable sample collection and high species richness (Figure 2). The curvature of the Shannon curve flattening with the increase of sequencing depth (Figure 2) could reflect the vast majority of microbial information in the intestinal tract. Compared to the similar uniformity in the two experimental groups, a milder curve in the control group showed a more uniform distribution of bacteria (Figure 2).

### 3.6 Diversity analysis

**Figure 3:**
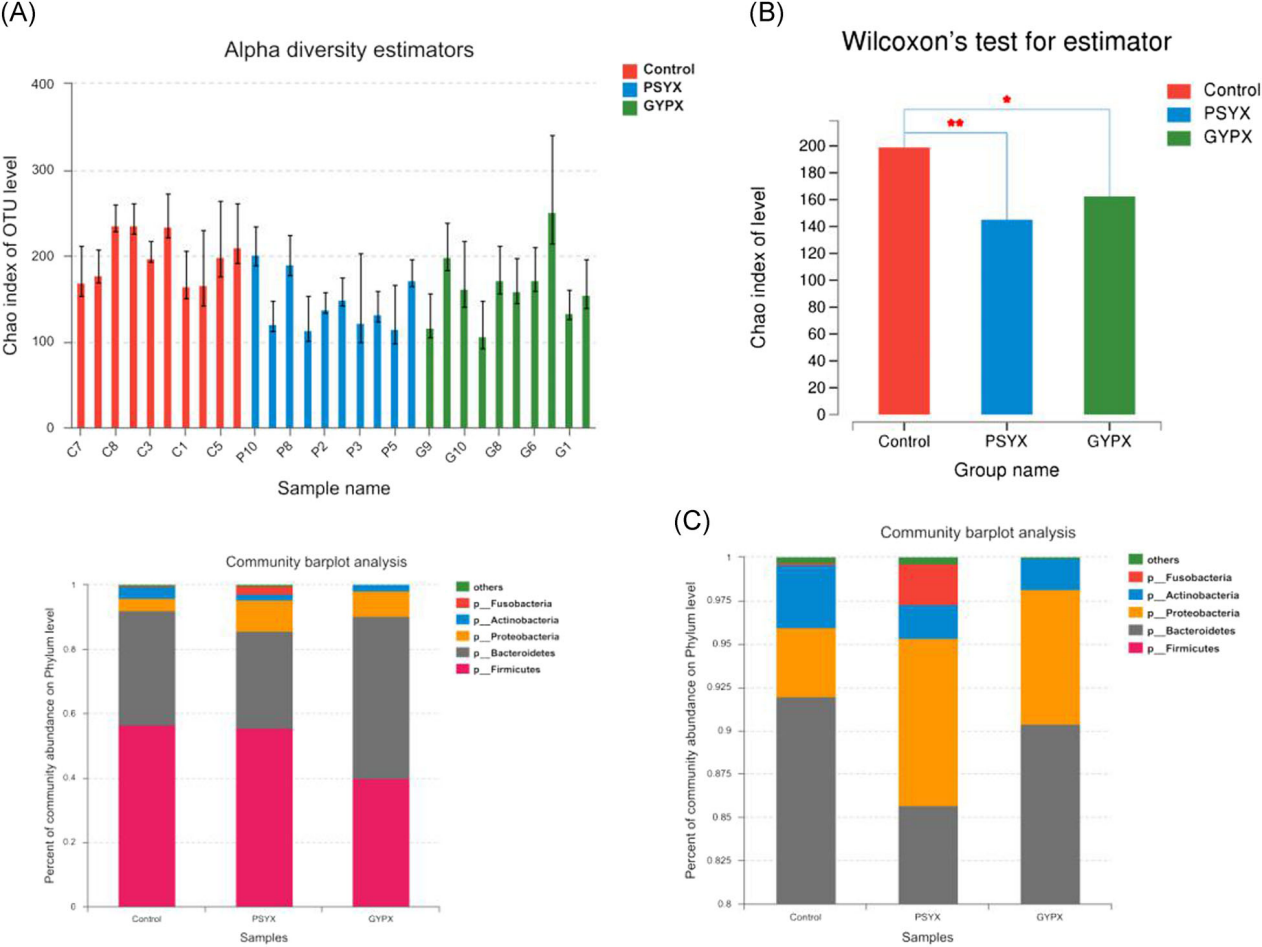
A, The Chao index of the control group and the experimental groups. B, Wilcoxon's test of Chao index of the control group and the experimental groups. C, Histogram of the phylum of the control group and the experimental groups. OTU, operational taxonomic unit

The Chao index of OTU level of group C and group L and group S was decreasing which meant the biological coverage as well as the bacterial diversity of the groups was decreasing (Figure 3). Wilcoxon's test showed the bacterial diversity of stool samples of group C was the richest while group S was the poorest. The difference between group C and group S was significant (*p* < .01); the difference between group C and group L was statistically significant (*p* < .05; Figure 3).

### 3.7 Multilevel analysis of species composition and group differences

**Figure 4:**
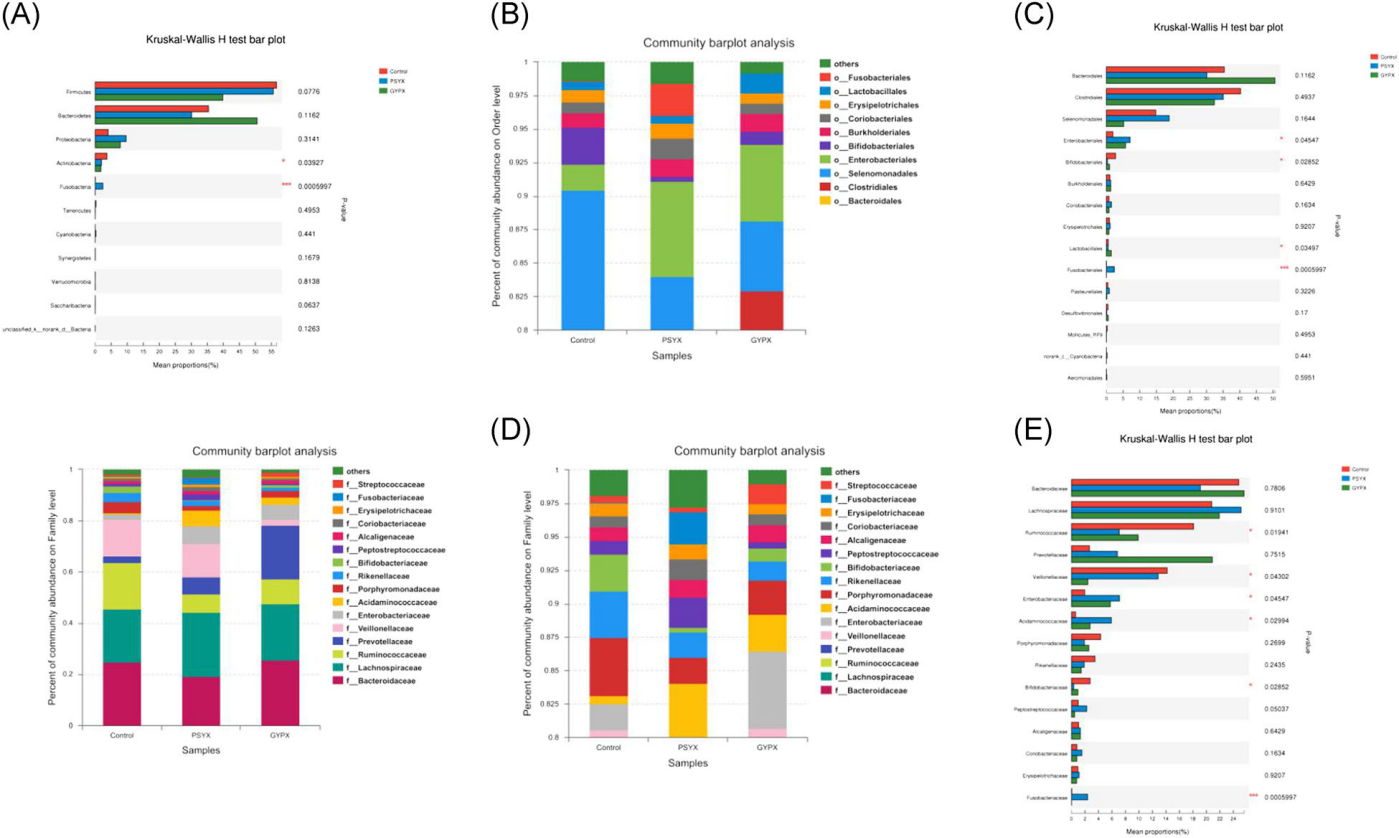
A, Wilcoxon's test of the phylum of the control group and the experimental groups. B, Histogram of the order of the control group and the experimental groups. C, Wilcoxon's test of the order of the control group and the experimental groups. D, Histogram of the family of the control group and the experimental groups. E, Wilcoxon's test of the family of the control group and the experimental groups

According to the histogram of phylum (Figures 3 and 4), we found that Actinobacteria accounted for 1.9% and 1.7% of the total amount of bacteria, respectively, in group S and group L, which was lower than that of group C accounting for 3.6% (*p* < .05). Fusobacteria (2.4%) in group S was higher than group C (0.05%) and group L (0.01%; *p* < .01). Enterobacteriales (7.1% and 5.8%) of the total amount of bacteria, respectively, in group S and group L, was higher than that of group C (2.0%; *p* < .05). Bifidobacteriales (0.3% and 1.0%) in group S and group L was lower than that of group C (2.8%; *p* < .05). Lactobacillae (1.5%) in group L  was higher than group C (0.6%) and group S (0.6%; *p* < .05). Fusobacteriales (2.4%) in group S was higher than group C (0.05%) and group L (0.01%; *p* < .01). The difference of the Bacteroidales, Clostridiales, Selenomonadales, Burkholderiales, Coriobacteriales, and Erysipelotrichales in the control group and experimental groups showed no statistical significance (*p* > .05; Figure 4).

Ruminococcaceae (7.1% and 9.9%) in group S and group L was lower than group C (18.1%; *p* < .05). Veillonellaceae (12.8% and 2.4%) in group S and group L was lower than group C (14.2%; *p* < .05). Enterobacteriaceae (7.1% and 5.8%) in group S and group L was higher than group C (2.0%; *p* < .05). Acidaminococcaceae (5.9% and 2.8%) in group S and group L was higher than group C (0.6%; *p* < .05). Bifidobacteriaceae (0.3% and 1.0%) in group S and group L was lower than group C (2.8%; *p* < .05). Fusobacteriaceae (2.4%) was higher than group C (0.05%) and group L (0.01%; *p* < .01). The difference of the Bacteroidales, Lachnospiraceae, Prevotellaceae, Porphyromonadaceae, Rikenellaceae, Peptostreptococcaceae, Alcaligenaceae, Coriobacteriaceae, Erysipelotrichaceae, and Streptococcaceae in the control group and experimental groups showed no statistical significance (*p* > .05; Figure 4).

### 3.8 Discriminant analysis of multilevel species difference

**Figure 5:**
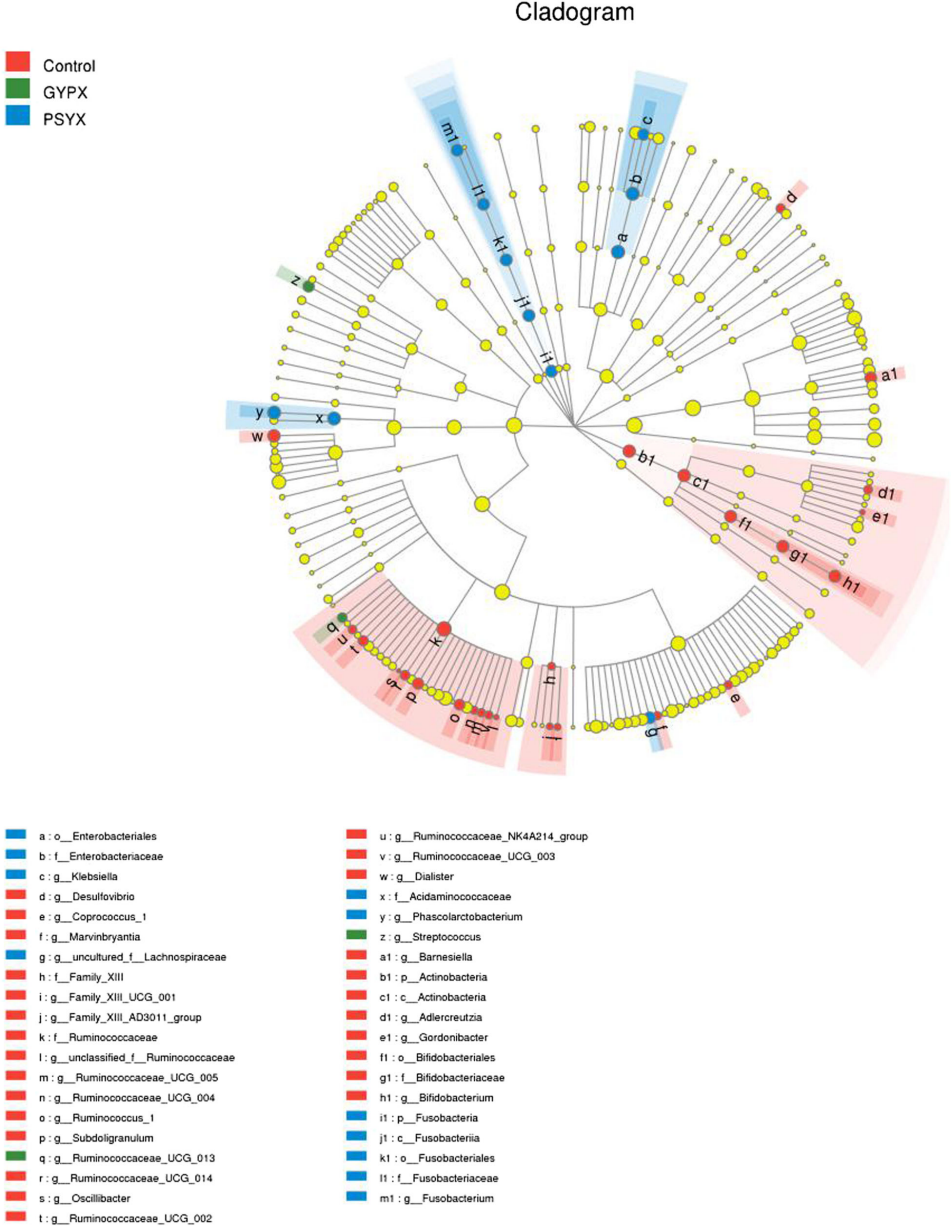
A, LEfSe of the control group and the experimental groups. B, PLS‐DA of the experimental groups, and the normal control group. PLS‐DA, partial least squares discriminant analysis

It was found that the Actinobacteria in group C and Fusobacteria in group S had specificity on the phylum level and class level. Besides this, there was a specificity of Bifidobacteriales in group C; and of Fusobacteriales and Enterobacteriales in group S on the order level; and on Ruminococcaceae and Bifidobacteriaceae in group C; and on Acidaminococcaceae, Enterobacteriaceae, and Fusobacteriaceae in group S on the family level; and on Dialister, Ruminococcaceae, Subdoligranulum, Marvinbryantia, Coprococcus, Bifidobacterium, Gordonibacter, Barnesiella, Adlercreutzia, Desulfovibrio, and Oscillibacter in group C; and on Fusobacterium, Klebsiella, and Phascolarctobacterium in group S; and on Streptococcus in group L on the genus level (Figure 5).

### 3.9 PLS‐DA

PLS‐DA is used to deal with the classification and discrimination that could effectively distinguish between intergroup observations. We could find that the difference between groups was greater than those within groups. The calculation method of the projection showed that the distribution of the three groups was relatively concentrated, and there was an interval between the groups while the difference between the three groups were obvious (Figure 5).

## 4 DISCUSSION

IBS is one of the most common gastrointestinal disorders.^21^ The surface has mainly *Escherichia coli* and Enterococcus, and the middle layer mainly bacillus, which is mainly Bifidobacterium and Lactobacillus.^22^ Some studies have found that there is a significant decrease in intestinal colonization resistance (the ratio of Bifidobacterium to Enterobacter [B/E]) in the feces of patients with IBS‐D, which refers to the increase of enterobacter, the decrease of bifidobacteria and lactobacillus,^18^ the increase of the Firmicutes to Bacteroidetes ratio (FBR),^23^ and the significant decreasing quantity of aerobic bacteria in the distal colonic mucosa.^24^

At present, the most common treatment method for patients with IBS‐D is probiotics. Sinn et al.^25^ found that after 4 weeks of treatment with acidophilus, the clinical symptoms including abdominal pain and abdominal discomfort were significantly improved. However, Simren et al.^26^ showed that the improvement of clinical symptoms has no significant curative effect after taking a complex probiotic preparation. Rousseaux et al.^27^ put forward the inference that probiotics were involved in regulating intestinal sensitivity that lactobacillus acidophilus could induce the expression of opioid receptors and cannabinoid receptors on intestinal mucosa epithelial cells. The mainstream medical treatment of IBS for probiotics can be summarized as follows^28^: probiotics could restrict the growth of pathogenic bacteria; probiotics could closely combine with intestinal mucosa to form a biological protective barrier to protect against the invasion and destruction of pathogenic bacteria; probiotics can inhibit the proliferation of pathogenic bacteria.

TCM has a unique set of syndrome differentiation systems for treating diarrhea IBS. Some scholars^29^ pointed out that IBS‐D could be caused by the liver, spleen, and kidney. A research study^30^ established the database syndromes of IBS‐D from 1989 to 2012, while the spleen–kidney‐yang deficiency and liver‐depression and spleen‐deficiency were the most common syndromes.

The current study found that there is a close interaction and influence between Chinese herbal medicine and intestinal flora so that Chinese herbal medicine has a good therapeutic effect on IBS‐D.^31^ Some studies^32^, ^33^ have confirmed that *Ginkgo biloba* compounds have a good bacteriostatic effect on common pathogenic bacteria such as *E. coli* in vitro. In addition, chicory fructose could increase the number of lactobacillus and Bifidobacterium, and chicory oligosaccharides could reduce the number of Salmonella and *E. coli* while the mechanism may be that the normal flora could be fermented by chicory fructose and oligosaccharides.^34^, ^35^, ^36^, ^37^ In this study, the dilutive curves generated by OUT^38^ tend to be flat, which reflects that the number of 30 samples in the experiment is reasonable to basically represent fecal bacteria in IBS‐D patients. Furthermore, the wide and gentle rank‐abundance curve indicates the high diversity and the uniform distribution of the species, which also reflects the reliability of the experiment.

### 4.1 Comparison of microbial diversity

Most studies on the relationship between IBS and intestinal microecology indicate that IBS has a decrease in intestinal flora diversity.^39^ In this experiment, we found that the diversity of fecal flora in IBS‐D group significantly reduce, which matches up with other studies.^40^ Besides this, the diversity of fecal flora was of the order: control group > liver‐depression and spleen‐deficiency group > spleen–kidney‐yang deficiency group. The diversity and richness of the microflora guarantee the stability of the microecosystem, ensure the intestinal resistance to bacteria, and also ensure the immune function of the intestinal tract which is reduced in the IBS‐D group. So, the brain–gut axis has more influence on the symptoms of the abdomen in the course of IBS, which makes for the difference between spleen–kidney‐yang deficiency and liver‐depression and spleen‐deficiency.

### 4.2 Characteristics of bacterial flora in IBS and healthy population

Compared with the control group, the most dominant bacteria in the IBS group are Firmicutes, Bacteroides, and Proteobacteria, which are consistent with most of the experimental results.^41^ Studies^42^ indicated that the two groups of bacteria play an important role in the process of human metabolism: Bacteroidetes carbohydrate metabolic processes are involved in the human body, the thick wall door is involved in the human body energy absorption process, when there is dysbacteriosis, the thick wall increases with the bacteroidetes bacteria door reduction can increase the incidence of obesity. In this experiment, we found that the number of Firmicutes was of the order: control group > spleen–kidney‐yang deficiency group > liver‐depression and spleen‐deficiency group, while the amount of Bacteroidetes had a significant increase in liver‐depression and spleen‐deficiency group. Compared with the liver‐depression and spleen‐deficiency group, B/E in the spleen–kidney‐yang deficiency group was lower, and the value of IBS groups was lower than 1 which was consistent with foreign research studies.^43^ The B/E value is the ratio of Bifidobacterium to Enterobacterium regarded as the colonization resistance proposed in 1971 by professor Van der Waaij, which is the ability of the mechanism whereby the intestinal microbiota protects itself against incursion by new and often harmful microorganisms.^44^ If the value of B/E is greater than 1, the intestinal implant resistance is normal, otherwise, it indicates a decrease of colonization resistance.^45^ The normal flora of the intestinal tract inhibits the reproduction of foreign bacteria.^46^ Some reports showed that the general trend in IBS patients of intestinal flora in China was a decline in lactobacillus and bifidobacterium, an ascent in enterobacterium, and no significant difference in bacteroides and coccobacillus while the trend of IBS in the rest of the world showed the decrease of bifidobacterium, and the increase of bacteroides and no significant change of lactobacillus and enterobacter.^47^ We found that this study was similar to those in both domestic and foreign countries with decrease of bifidobacterium, increase of enterobacter.

### 4.3 Comparison of bacterial characteristics

Bifidobacterium is one of the most familiar probiotics, which has the function of maintaining the normal bacteria flora balance of the intestinal tract, preventing constipation, diarrhea, and gastrointestinal dysfunction and has been widely used. Ait‐Belgnaoui et al.^48^ found that *Bifidobacterium longum* and *Lactobacillus helveticus* synergistically suppress stress‐related visceral hypersensitivity through hypothalamic–pituitary–adrenal axis modulation. Gu et al.^49^ pointed out that *B. longum* might reduce IL‐18 and IL‐1β expressions by inhibiting NLRP3 inflammasome to reduce the visceral hypersensitivity of PI‐IBS. In this study, the bifidobacteria in the control group accounted for 2.8%, which was significantly higher than that of spleen–kidney‐yang deficiency group and liver‐depression and spleen‐deficiency group; furthermore, the Bifidobacterium ratio of liver‐depression and spleen‐deficiency group is higher than spleen–kidney‐yang deficiency group. In the theory of TCM, the pathogenesis of spleen–kidney‐yang deficiency is longer, and the long period of microecological environment disorder makes the number of bifidobacteria rare.

Recently, several researchers^50^ have reported that the level of Fusobacterium is significantly elevated in human colorectal adenomas and carcinomas compared to that in adjacent normal tissue. The metabolites of fusobacterium contain butyric acid, which could promote visceral hypersensitivity via enteric glial cell‐derived nerve growth factor in an IBS‐like model.^51^, ^52^ It was found that compared with the normal group, the enterobacter of IBS patients increased significantly and the intestinal colonization resistance was significantly reduced.^53^ In addition to the above three kinds of bacteria, there is a significant difference in Ruminococcaceae, Acidaminococcaceae, and Veillonellaceae. Tana et al.^54^ found that the quality of Ruminococcaceae and lactobacillus and the level of acetic acid, propionic acid, and total organic acids in IBS patients was significantly increased, which showed the abnormal presence of fecal bacteria and organic acids in IBS patients. Therefore, it is speculated that the above‐mentioned bacteria which are caused by excessive intestinal gas production, the destruction of intestinal osmotic pressure, and intestinal immune function results in the occurrence and development of IBS.

In the course of the study, we found that Bifidobacterium, Fusobacterium, Enterobacterium, and Streptococcus were strongly specific to each group. It was proved that bifidobacterium has a good curative effect in IBS patients with long‐term clinical practice, and has been widely used in the clinic to improve the symptoms of IBS patients such as reducing the frequency of bowel movements and improving the fecal character. The level of Fusobacterium and Enterobacterium in spleen–kidney‐yang deficiency was significantly increased. Streptococcus was a suppurative strain which was more specific in liver‐depression and spleen‐deficiency. The bacteria may produce inflammatory factors to stimulate the intestinal mucosa and increase intestinal sensitivity. The above four bacteria may become iconic microflora because of their strong specificity. However, the mechanism still needs further research and exploration in the later stage.

## 5 CONCLUSION

It was confirmed by the bacterial level that the disorder of intestinal flora is one of the important mechanisms of IBS‐D. There is a correlation between intestinal flora and TCM syndromes in that the spleen–kidney‐yang deficiency and liver‐depression and spleen‐deficiency have their specific bacterial flora.

## CONFLICT OF INTERESTS

The authors declare that there are no conflict of interests.

## AUTHOR CONTRIBUTIONS

Guanqun Chao wrote the article and Shuo Zhang provided guidance.

## ETHICS STATEMENT

The study has been approved by the ethics committee of Zhejiang Chinese Medical University.

## Data Availability

The datasets used and/or analyzed during the current study are available from the corresponding author on reasonable request.
